# Editorial: Computational Methods in Predicting Complex Disease Associated Genes and Environmental Factors

**DOI:** 10.3389/fgene.2021.679651

**Published:** 2021-04-21

**Authors:** Yudong Cai, Jialiang Yang, Tao Huang, Minxian Wallace Wang

**Affiliations:** ^1^School of Life Sciences, Shanghai University, Shanghai, China; ^2^Geneis (Beijing) Co. Ltd., Beijing, China; ^3^Bio-Med Big Data Center, CAS Key Laboratory of Computational Biology, Shanghai Institute of Nutrition and Health, Chinese Academy of Sciences, Shanghai, China; ^4^Medical and Population Genetics Program, Broad Institute of MIT and Harvard, Cambridge, MA, United States

**Keywords:** computational method, complex disease, environmental factor, disease gene, genetic factor, epigenetic factor

With the advances of sequencing and experimental techniques, the molecular mechanisms of human Mendelian diseases have been more or less elucidated. However, there are also many complex diseases whose disease/pathology development involves the interaction of large numbers of biomolecules across multi-molecular levels including DNA, RNA, proteins, and methylation, as well as the impact of environmental and human lifestyle factors. The understanding of such diseases is one of the biggest challenges in modern biology and medical sciences. The progress in this field will shed light on complex disease pathology, prevention, prognosis, diagnosis, and treatment in a personalized manner.

In recent years, large amounts of data from human genome sequencing, metagenome sequencing, and information about the impact of environmental and lifestyle factors on complex diseases have been produced, collected, and stored in large scale databases such as the National Alzheimer's Coordinating Center (NACC) database, the database of Genotypes and Phenotypes (dbGaP) and UK Biobank. The large amount of data poses a big challenge, as well as a great opportunity, to reveal the secrets behind complex diseases using machine learning, statistics, and bioinformatics tools along with validation through experimental work. In fact, many computational studies have already been performed within this research area; however, most are focused on disease-associated factors at a single-molecular level, such as genetic factors, epigenetic factors, environmental factors, and so on. A more systematic study on the interactions among these factors, alongside experimental validation, might present a comprehensive view on the disease pathogenicity and thus may hold the key to truly understanding complex diseases.

In this special issue, there are 18 studies of complex diseases.

Li et al. compared the gene expression profiles between patients with heart failure (*n* = 177) and without heart failure (*n* = 136) using multiple feature selection strategies and identified 38 HF signature genes. Their results can facilitate the early detection of heart failure and can reveal its molecular mechanisms.

Liang et al. proposed a novel antiviral Drug Repositioning method based on minimizing Matrix Nuclear Norm (DRMNN). Experiments have shown that DRMNN is better than other algorithms in predicting which drugs are effective against influenza A virus. Within the 10 drugs most likely to be effective against H3N2 viruses, six drugs are reported to have some effect on the viruses.

Liu et al. recruited 20 patients undergoing cardiac surgery (10 with paroxysmal atrial fibrillation and 10 with persistent atrial fibrillation) and 10 healthy subjects. With proteomic analysis, they identified the differentially expressed proteins and investigated their roles in Atrial fibrillation (AF).

Li et al. developed a set of computational approaches integrating multiple machine-learning algorithms, including Monte Carlo feature selection (MCFS), incremental feature selection (IFS), and support vector machine (SVM), to identify gene expression characteristics on different phases of Myocardial infarction (MI). The functional enrichment analyses followed by protein-protein interaction analysis identified several hub genes (IL1R1, TLR2, and TLR4) which may be new diagnostic molecules for MI.

Su et al. described a convolutional neural network called F-S-Net that fused the information from multimodal medical images and used the semantic information contained within these images for glioma segmentation. F-S-Net was found to achieve a dice coefficient of 0.9052 and Jaccard similarity of 0.8280, outperforming several previous segmentation methods.

Wang et al. screened the genes associated with neuropathic pain (NP) using differential analysis along with random walk with restart (RWR). They discovered eight hub genes that were closely related to NP occurrence and development, which may help to provide potent theoretical basis for NP treatment.

Hao et al. integrated four gene expression datasets which collectively included 65 nasal polyp samples from Chronic rhinosinusitis with nasal polyps (CRSwNP) patients and 54 nasal mucosal samples from healthy controls. They identified 76 co-differentially expressed genes (co-DEGs, including 45 upregulated and 31 downregulated) in CRSwNP patients compared with the healthy controls. Protein-protein interaction (PPI) network analysis and real-time quantitative PCR (RT-qPCR) showed that seven genes might be crucial in CRSwNP pathogenesis.

Guan et al. constructed cell type-specific predictive models for autism spectrum disorder (ASD) based on individual genes and gene sets, respectively, to screen cell type-specific ASD-associated genes and gene sets. They found that the functions of genes with predictive power for ASD were different and the top important genes were distinct across different cells, highlighting the cell-type heterogeneity of ASD.

Zhu et al. proposed and compared 10 protein–protein interaction (PPI)-based computational methods to study the connections between diabetes and 254 diseases. They found that a method called DIconnectivity_eDMN performed the best in the sense that it inferred a disease rank (according to its relation with diabetes) most consistent with that by literature mining.

Zhang et al. analyzed the blood gene expression profiles of 73 Caucasian women with high and low bone mineral density (BMD). The WGCNA yielded three gene modules, including 26 lncRNAs and 55 mRNAs as hub genes in the blue module, 36 lncRNAs and 31 mRNAs as hub genes in the turquoise module, and 56 mRNAs and 30 lncRNAs as hub genes in the brown module. The mRNAs and lncRNAs identified in this WGCNA could be novel clinical targets in the diagnosis and management of osteoporosis.

Sun et al. proposed a mathematical model based on matrix decomposition, named MFMDA, to identify potential miRNA–disease associations by integrating known miRNA and disease-related data, similarities between miRNAs and between diseases. While most predicted miRNAs were confirmed by external databases of experimental literature, they also identified a few novel disease-related miRNAs for further experimental validation.

Wang et al. identify the key modules and hub genes related to the annulus fibrosus in intervertebral disc degeneration (IDD) through: (1) constructing a weighted gene co-expression network; (2) identifying key modules and hub genes; (3) verifying the relationships of key modules and hub genes with IDD; and (4) confirming the expression pattern of hub genes in clinical samples. They generated a comprehensive overview of the gene networks underlying annulus fibrosus in intervertebral disc degeneration.

Wang et al. proposed a new method called Matrix completion algorithm based on q-kernel information (QIMCMDA) for miRNA-disease association prediction. Its performance was significantly better than other commonly used technologies. QIMCMDA may become an excellent supplement in the field of biomedical research in the future.

Liu et al. proposed a novel network inference algorithm using Random Walk with Restart (RWRNET) that combined local and global topology relationships. The proposed method was compared with several state-of-the-art methods on the basis of six benchmark datasets and the results demonstrated the effectiveness of the proposed method.

An et al. evaluated the pharmacological effects of novel peptide drugs (P-ONE and P-TWO) at the small RNA (sRNA) level using an allergic rhinitis (AR) model. They found that sRNA target genes had a specific enrichment pattern and may contribute to the effects of the novel peptides.

Gao et al. identified orphan genes in balanced and unbalanced *Arabidopsis thaliana* gene datasets. They compared several ensemble models and found that SMOTE-ENN-XGBoost model, which combined over-sampling and under-sampling algorithms with XGBoost, achieved higher predictive accuracy than the other balanced algorithms with XGBoost models. Thus, SMOTE-ENN-XGBoost provided a theoretical basis for developing evaluation criteria for identifying orphan genes in unbalanced and biological datasets.

Wei et al. developed a machine learning method to classify colon and rectal cancer into three immune subtypes named High-Immunity Subtype, Medium-Immunity Subtype, and Low-Immunity Subtype, respectively. A prognostic signature of six genes (CERCAM, CD37, CALB2, MEOX2, RASGRP2, and PCOLCE2) was identified by the multivariable COX analysis, which was further used to develop an accurate model to predict the prognosis of colon and rectal cancer patients.

Chidambaran et al. recruited 171 adolescents (14.5 ± 1.8 years, 75.4% female) undergoing spine fusion and tested ranked deciles of 1,336 prioritized genes for increased representation of variants associated with chronic postsurgical pain (CPSP). Penalized regression (LASSO) selected 20 variants for calculating weighted polygenic risk scores (PRS). Systems biology guided PRS improved predictive accuracy of CPSP risk in a pediatric cohort.

In recent years, there are more and more studies of complex diseases using computational methods on multi omics data. By integrating genetic factors, epigenetic factors, environmental factors, and so on, the underlying mechanisms of complex diseases may be revealed and we may find the cures.

## Author Contributions

TH wrote the editorial and all authors have approved the submission.

## Conflict of Interest

JY is the Vice President of Geneis (Beijing) Co. Ltd. The remaining authors declare that the research was conducted in the absence of any commercial or financial relationships that could be construed as a potential conflict of interest.

